# Cross-sectional and longitudinal associations of apolipoprotein A1 and B with glycosylated hemoglobin in Chinese adults

**DOI:** 10.1038/s41598-022-06829-w

**Published:** 2022-02-17

**Authors:** Hongli Dong, Wenqing Ni, Yamin Bai, Xueli Yuan, Yan Zhang, Hongmin Zhang, Yuanying Sun, Jian Xu

**Affiliations:** 1 Scientific Education Section and Department of Child Healthcare, Affiliated Maternity & Child Health Care Hospital of Nantong University, Nantong, China; 2 Department of Elderly Health Management, Shenzhen Center for Chronic Disease Control, Shenzhen, China; 3 Center for Chronic and Non-Communicable Diseases Control and Prevention, Chinese Center for Disease Control and Prevention, Beijing, China

**Keywords:** Cardiology, Endocrinology, Risk factors

## Abstract

Apolipoproteins exert a key role on glucose metabolism; however, scarce data have examined the relationship between apolipoproteins and glycated haemoglobin (HbA1c) in Chinese adults. This study determined the cross-sectional and longitudinal associations of serum Apolipoprotein A1 (ApoA1), Apolipoprotein B (ApoB) and the ApoB/A1 ratio with HbA1c in Chinese adults. A total of 1448 subjects (584 men and 864 women) aged 54.8 years were included in a baseline survey, and the concentrations of Apo and HbA1c were measured. A total of 826 participants were followed up approximately once after 3.94 ± 0.62 years. In cross-sectional analysis, serum ApoA1 was inversely associated with HbA1c, while ApoB and the ApoB/A1 ratio were positively associated with HbA1c. After further adjusting for the potential covariates, a higher ApoA1 was associated with lower HbA1c (Quartile 4 [Q4] vs. Q1 = 5.673% vs. 5.796%, *P*-trend = 0.014). In contrast, positive association of ApoB concentration and the ApoB/A1 ratio with HbA1c level were showed (Q4 vs. Q1 = 5.805% vs. 5.589% for ApoB; Q4 vs. Q1 = 5.841% vs. 5.582% for ApoB/A1 ratio). The longitudinal results showed no significant associations of ApoA1, ApoB levels and the ApoB/A1 ratio with HbA1c changes (all *P*-trends > 0.05). Path analysis suggested that body mass index did not have mediating effect on Apo-HbA1c association. Our findings revealed that higher ApoA1, lower ApoB concentrations and the ApoB/A1 ratio were associated with lower HbA1c level in Chinese adults.

## Introduction

Glycated haemoglobin (HbA1c), an integrated measure of circulating blood glucose levels during the previous 2 to 3 months, is considered as a gold standard for long period follow-up of blood glycemic control^1,2^. Elevated HbA1c level is one of the predominant risk factors for diabetes and its complications^3^. Stratton et al.^4^ found that the reduction in updated mean HbA1c level was beneficial to the reduction in risk of any diabetes-related end point and deaths. Therefore, it is urgent to explore the novel modifiable factors of HbA1c for the improvement of diabetes and its complications.

Apolipoproteins, a main protein part of lipoproteins, play an important role in the pathological process of type 2 diabetes mellitus^5,6^. Apolipoprotein A1 (ApoA1) is the major lipoprotein associated with high-density lipoprotein cholesterol (HDL-C) and exert a key part in the transfer of cholesterol from the periphery to the liver in the circulation^7^. Apolipoprotein B (ApoB) is the major protein part of low-, intermediate-, and very low-density lipoproteins^8^. In vitro and animal studies have demonstrated anti- or pro-diabetic effects of ApoA1 and ApoB^9–11^, observational studies aimed at exploring the association of ApoA1 and ApoB with HbA1c level found the inverse or null association with ApoA1^12–14^ and positive association with ApoB^13^. These studies suggest that ApoA1 and ApoB may play important roles in maintaining circulating HbA1c level. Previous study indicated that the levels of ApoA, ApoB, and lipoprotein were lower in Chinese adults than in Caucasians^15^, however, scarce data is available for Chinese populations who differ from their Western counterparts in concentrations of lipoprotein^12–14^. Additionally, the different prevalence of obesity between Western and Chinese populations may have impact on the Apo-HbA1c association because body fat can affect the body's glucometabolism^16,17^. Therefore, the Apo-HbA1c association in Chinese populations remains unclear.

Obesity is an important risk factor in maintaining circulating HbA1c level^18^. Previous study demonstrated a significant correlation between Apo levels and obesity^19^. No study, however, examined whether or not the association between Apos and HbA1c is mediated by obesity.

The present study assessed the cross-sectional and longitudinal associations of ApoA1, ApoB levels and the ApoB/A1 ratio with HbA1c concentrations, and examined the mediating effects of body mass index (BMI) in the Apo-HbA1c association in middle-aged and elderly Chinese.

## Materials and methods

### Study subjects

**Figure 1:**
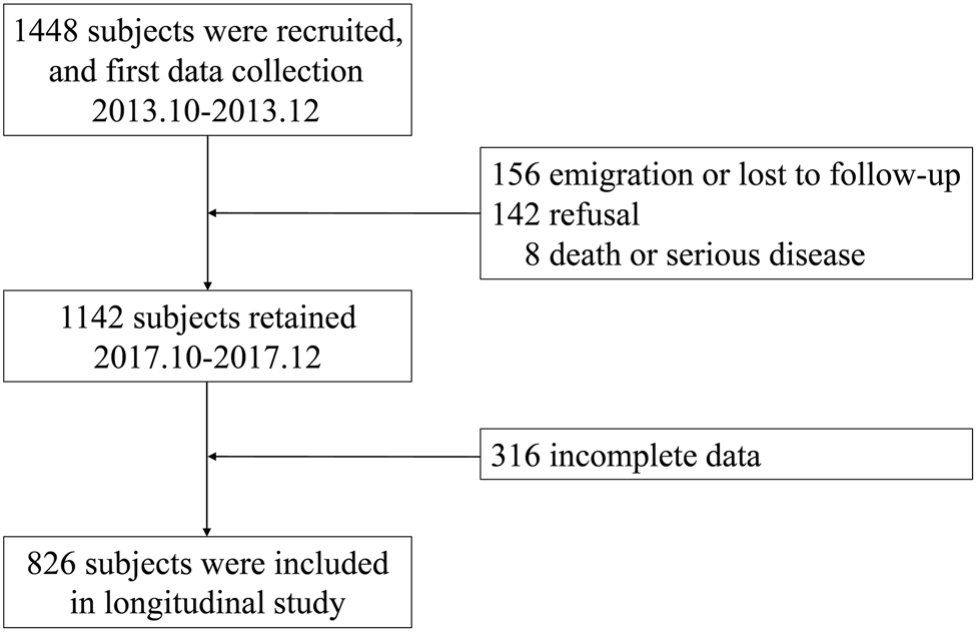
Flow chart of study subjects.

A community-based longitudinal study was conducted in Shenzhen, China, during a period from October 2013 to December 2017. 1448 participants aged 54.8 years from October to December 2013 were recruited to complete a baseline survey. ApoA1, ApoB, lipids, and HbA1c concentrations were examined at baseline. The subjects were followed up once in October to December 2017. During the follow-up period, subjects were excluded (n = 306) according to the following pre-defined criteria: (1) emigration or lost to follow-up (n = 156); (2) refusal (n = 142); (3) death or serious disease (n = 8). Finally, 1142 subjects completed their follow-up. We further excluded those with incomplete data (n = 316). A total of 826 subjects were included in the follow-up analyses (Fig. 1). HbA1c were examined in this follow-up. The present study was approved by the Shenzhen Center for Chronic Disease Control Human Ethics Committee (No. 20130411) and therefore performed in accordance with the 1964 Declaration of Helsinki and its later amendments. Written informed consent was obtained from all participants.

### Questionnaire interview and laboratory assays

Detailed sociodemographic characteristics and health parameters were collected by a structured questionnaire^20^ including age, marriage, sex, education level, alcohol consumption (current drinker or non-drinker), cigarette smoking (current smoker or non-smoker), exercise (sitting, light, moderate, strenuous), lipid-lowering drugs and hypoglycemic medication use. The body height and weight of the participants were measured, and BMI was calculated. Vein blood samples of the participants were collected after 10–14 h of fasting. Serum triglyceride (TG), total cholesterol (TC), HDL-C and low density lipoprotein cholesterol (LDL-C) concentrations were determined using commercial reagents (Olympus System Reagents, Olympus Diagnostica, Ireland) in an autoanalyzer (Olympus AU400 System, Tokyo, Japan). The HbA1c level in the red blood cells was measured by a Bole glycated hemoglobin D-10 kit on a Bole glycated hemoglobin analyzer D-10. Serum ApoA1 and ApoB concentrations were measured based on standardized operation. Briefly, serum sample (3 µL) were mixed with the reagent (240 µL) composed of polyethylene glycol (30 g/L) and phosphate buffer (0.05 mol/L, pH = 7.0). The mixture was vortexed for 2–3 min and then incubated for 5 min at 37 °C. The concentrations of ApoA1 and ApoB were detected using the polyethylene glycol-enhanced immunology turbidimetric assay in a 7600-010 automatic analyzer (Hitachi, Japan). The CVs of ApoA1 and ApoB were 3.7 and 4.5%, respectively. ApoB/A1 ratio was calculated.

### Statistical analyses

Categorical- and continuous-variables were described using frequencies (percentages) and means (± SD) in both women and men. The mean difference in the continuous variables and the proportion difference among categorical variables were evaluated using Student’s t-test and Chi-square test, respectively. According to ApoA1, B levels and the ApoB/A1 ratio, the participants were divided into quartiles, respectively. The lowest quartile defined as the reference. The HbA1c level for the 2nd–4th quartiles of Apo were described using means and standard error (SE). The mean differences of HbA1c level and the trends in quartiles of Apo were tested using multivariate–analyses of covariance (ANCOVAs). The pair-wise comparisons were examined among quartiles by the Bonferroni test. Univariate analysis was conducted in Model 1. Sex and age were adjusted in Model 2. BMI, education, marriage, exercise, cigarette smoking, alcohol consumption, hypoglycaemic agent, and lipid-lowering drugs use were further adjusted in Model 3. The mediating effects of BMI on Apo-HbA1c association were evaluated using path analyses^21^. Standardized regression coefficients were assessed in each path. SPSS Statistics 21.0 (IBM SPSS Statistics, USA) and SPSS AMOS21.0 (IBM Corporation, USA) were performed to analyze the data. Two-tailed *P* < 0.05 was considered statistically significant.

## Results

### Characteristics of participants

As shown in Table 1, the mean age of men and women were 55.1 and 54.6 years, respectively. Lower BMI (23.84 kg/m^2^ vs. 24.34 kg/m^2^), TG (1.42 mmol/L vs. 1.64 mmol/L), ApoB (0.99 g/L vs. 1.02 g/L), and the ApoB/A1 ratio (0.65 vs. 0.77) and higher TC (5.04 mmol/L vs. 4.97 mmol/L), HDL-C (1.41 mmol/L vs. 1.31 mmol/L) and ApoA1 (1.56 g/L vs. 1.43 g/L) were observed in women than in men (all *P* < 0.05).

**Table 1:** The baseline characteristics of the study participants.

Variables	Men (n = 584)	Women (n = 864)	*P*^a^ value
Age, years	55.1 ± 12.00	54.6 ± 11.44	0.406
BMI, kg/m^2^	24.34 ± 3.07	23.84 ± 3.05	**0.002**
**Education level, n (%)**			**< 0.001**
Junior high school	44 (7.5)	143 (16.6)	
High school	280 (47.9)	440 (50.9)	
College degree or above	260 (44.5)	281 (32.5)	
**Marriage, n (%)**			**0.005**
Married	562 (96.2)	795 (92.0)	
Unmarried	4 (0.7)	16 (1.9)	
Divorce/widowed	18 (3.1)	53 (6.1)	
**Exercise, n (%)**			**0.008**
Sitting	423 (72.4)	624 (72.2)	
Light	103 (17.6)	152 (17.6)	
Moderate	34 (5.8)	74 (8.6)	
Strenuous	24 (4.2)	14 (1.6)	
Smoker, n (%)	201 (34.4)	22 (2.5)	**< 0.001**
Alcohol drinker, n (%)	131 (22.4)	49 (5.7)	**< 0.001**
Lipid-lowering drugs user, n (%)	42 (7.2)	60 (6.9)	0.857
Hypoglycaemic agent user, n (%)	19 (3.3)	26 (3.0)	0.793
TC (mmol/L)	4.97 ± 0.77	5.04 ± 0.68	**0.042**
TG (mmol/L)	1.64 ± 1.24	1.42 ± 0.92	**< 0.001**
HDL-C (mmol/L)	1.31 ± 0.52	1.41 ± 0.37	**< 0.001**
LDL-C (mmol/L)	2.90 ± 0.71	2.87 ± 0.66	0.343
ApoA1, g/L	1.43 ± 0.29	1.56 ± 0.26	**< 0.001**
ApoB, g/L	1.02 ± 0.24	0.99 ± 0.23	**0.032**
ApoB/A1 ratio	0.77 ± 0.47	0.65 ± 0.19	**< 0.001**
HbA1c, %	5.66 ± 0.66	5.71 ± 0.65	0.198

*Apo* Apolipoprotein, *BMI* body mass index, *HbA1c* Glycated haemoglobin, *HDL-C* high density lipoprotein cholesterol, *LDL-C* low density lipoprotein cholesterol, *TC* total cholesterol, *TG* triglycerides. ^a^*P* values were calculated by Student’s t test for the continuous variables and Chi-square test for categorical variables.

Significant values are in bold.

### Partial correlation analysis

Table 2 showed the inverse relationship between ApoA1 and HbA1c in both models 1 and 2 in partial correlation analysis. In contrast, positive association of ApoB concentration and the ApoB/A1 ratio with HbA1c level were showed (Table 2).

**Table 2:** Relationships between serum apolipoprotein levels and HbA1c^a^.

	Model 1		Model 2	
	*r′*	*P*	*r′*	*P*
ApoA1	− 0.146	**< 0.01**	− 0.148	**< 0.01**
ApoB	0.142	**< 0.01**	0.144	**< 0.01**
ApoB/A1 ratio	0.333	**< 0.01**	0.332	**< 0.01**

Abbreviations were shown in Table 1.

^a^ Partial correlation analysis, Model 1 adjusted for age and gender. Model 2 adjusted for age, gender, BMI, education, marriage, exercise, cigarette smoking, alcohol consumption, hypoglycaemic agent, and lipid-lowering drugs use.

Significant values are in bold.

### Associations of ApoA1, ApoB, and the ApoB/A1 ratio with HbA1c level

In cross-sectional analysis, serum ApoA1 showed an inverse association with HbA1c, while ApoB and the ApoB/A1 ratio exhibited positive association with HbA1c (Table 3). In Model 1 with univariate analysis, ApoA1 was inversely related to HbA1c (*P*-trend = 0.046), whereas positive associations were observed for ApoB level and the ApoB/A1 ratio (*P*-trend < 0.001). With adjustment for sex and age in Model 2, ApoA1 concentration was inversely associated with HbA1c level (quartile 4 [Q4] vs. Q1 = 5.671% vs. 5.801%, *P*-trend = 0.010). In contrast, ApoB concentration and the ApoB/A1 ratio were positively associated with HbA1c level (Q4 vs. Q1 = 5.808% vs. 5.588% for ApoB; Q4 vs. Q1 = 5.839% vs. 5.584% for ApoB/A1 ratio). After further adjusting for the other potential covariates in Model 3, a higher ApoA1 concentration was associated with lower HbA1c level (Q4 vs. Q1 = 5.673% vs. 5.796%, *P*-trend = 0.014). In contrast, the positive associations of ApoB concentration and the ApoB/A1 ratio with HbA1c level were showed (Q4 vs. Q1 = 5.805% vs. 5.589% for ApoB; Q4 vs. Q1 = 5.841% vs. 5.582% for ApoB/A1 ratio). Additionally, we found null associations of ApoA1, ApoB levels and the ApoB/A1 ratio with HbA1c changes across the three models (all *P*-trends > 0.05) (Table 4).

**Table 3:** Mean HbA1c level according to quartiles of apolipoprotein in all participants (mean ± SE).

Variables		Quartiles by apolipoproteins				*P*-diff	*P*-trend
		Q1	Q2	Q3	Q4		
**ApoA1**							
n		362	361	357	368		
HbA1c	Model 1	5.783 ± 0.034	**5.640 ± 0.034**^**a**^	**5.646 ± 0.034**^**a**^	5.679 ± 0.034	**0.011**	**0.046**
	Model 2	5.801 ± 0.034	**5.636 ± 0.034**^**a**^	**5.640 ± 0.034**^**a**^	**5.671 ± 0.034**^**a**^	**< 0.001**	**0.010**
	Model 3	5.796 ± 0.033	**5.638 ± 0.033**^**a**^	**5.640 ± 0.033**^**a**^	5.673 ± 0.033	**0.002**	**0.014**
**ApoB**							
n		365	368	357	358		
HbA1c	Model 1	5.576 ± 0.034	5.626 ± 0.034	**5.732 ± 0.034**^**a**^	**5.819 ± 0.034**^**bc**^	**< 0.001**	**< 0.001**
	Model 2	5.588 ± 0.033	5.631 ± 0.033	**5.725 ± 0.034**^**a**^	**5.808 ± 0.034**^**bc**^	**< 0.001**	**< 0.001**
	Model 3	5.589 ± 0.033	5.631 ± 0.032	**5.728 ± 0.033**^**a**^	**5.805 ± 0.033**^**bc**^	**< 0.001**	**< 0.001**
**ApoB/A1 ratio**							
n		362	362	362	362		
HbA1c	Model 1	5.578 ± 0.034	5.614 ± 0.034	**5.719 ± 0.034**^**a**^	**5.838 ± 0.034**^**bc**^	**< 0.001**	**< 0.001**
	Model 2	5.584 ± 0.033	5.621 ± 0.033	5.704 ± 0.033	**5.839 ± 0.033**^**bcd**^	**< 0.001**	**< 0.001**
	Model 3	5.582 ± 0.033	5.630 ± 0.033	5.696 ± 0.033	**5.841 ± 0.033**^**bcd**^	**< 0.001**	**< 0.001**

*Apo* apolipoprotein, *HbA1c* glycosylated hemoglobin, *Q* quartile. *P*-Diff: Multiple comparison among quartiles. Model 1 was univariate analysis. Model 2 was adjusted for sex, age. Model 3 further adjusted for BMI, education, marriage, exercise, cigarette smoking, alcohol consumption, hypoglycaemic agent, and lipid-lowering drugs use. ^a^*P* < 0.05 compared with Q1. ^b^*P* < 0.001 compared with Q1. ^c^*P* < 0.001 compared with Q2. ^d^*P* < 0.05 compared with Q3.

Significant values are in bold.

**Table 4:** Changes in HbA1c level over ~ 4 year by quartiles of apolipoprotein in all participants (mean ± SE).

Variables		Quartiles by apolipoproteins				*P*-diff	*P*-trend
		Q1	Q2	Q3	Q4		
**ApoA1**							
n		208	203	211	204		
HbA1c	Model 1	0.028 ± 0.047	− 0.126 ± 0.048	− 0.049 ± 0.047	0.026 ± 0.048	0.069	0.732
	Model 2	0.029 ± 0.047	− 0.126 ± 0.048	− 0.050 ± 0.047	0.026 ± 0.048	0.068	0.760
	Model 3	0.036 ± 0.047	− 0.118 ± 0.048	− 0.054 ± 0.047	0.015 ± 0.048	0.091	0.998
**ApoB**							
n		209	205	207	205		
HbA1c	Model 1	− 0.024 ± 0.047	− 0.014 ± 0.048	− 0.038 ± 0.048	− 0.045 ± 0.048	0.968	0.683
	Model 2	− 0.022 ± 0.048	− 0.014 ± 0.048	− 0.038 ± 0.048	− 0.046 ± 0.048	0.964	0.656
	Model 3	− 0.027 ± 0.047	− 0.015 ± 0.048	− 0.031 ± 0.047	− 0.048 ± 0.048	0.969	0.708
**ApoB/A1 ratio**							
n		206	207	207	206		
HbA1c	Model 1	− 0.042 ± 0.048	− 0.057 ± 0.048	0.009 ± 0.048	− 0.031 ± 0.048	0.786	0.640
	Model 2	− 0.041 ± 0.048	− 0.057 ± 0.048	0.008 ± 0.048	− 0.031 ± 0.048	0.796	0.653
	Model 3	− 0.050 ± 0.048	− 0.065 ± 0.047	0.019 ± 0.048	− 0.024 ± 0.048	0.621	0.455

*Apo* apolipoprotein, *HbA1c* glycosylated hemoglobin, *Q* quartile. *P*-Diff: Multiple comparison among quartiles. Model 1 was univariate analysis. Model 2 was adjusted for sex, age. Model 3 further adjusted for BMI, education, marriage, exercise, cigarette smoking, alcohol consumption, hypoglycaemic agent, and lipid-lowering drugs use.

### Associations of serum lipids levels with HbA1c level

As shown in Supplementary Table S1, a higher serum HDL-C was associated with lower HbA1c. In contrast, higher serum TG and TC concentration were associated with higher HbA1c level across the three models. Null association between serum LDL-C with HbA1c level was detected. At follow-up, no significant associations were observed between ApoA1, ApoB levels and the ApoB/A1 ratio with HbA1c changes (all *P*-trends > 0.05) (Supplementary Table S2).

### Path analysis

**Figure 2:**
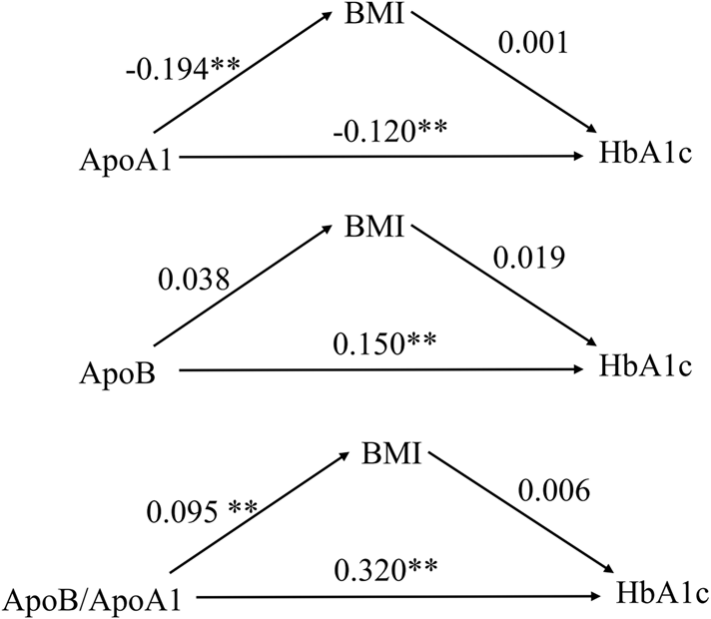
Path analyses of associations of ApoA1, ApoB levels, the ApoB/A1 ratio, mediator (BMI) with HbA1c in subjects. *Apo* Apolipoprotein, *BMI* body mass index, *HbA1c* glycated haemoglobin; **P < 0.05.

Path analysis was used to assess whether BMI mediated the associations of ApoA1, ApoB levels and the ApoB/A1 ratio with HbA1c. As shown in Fig. 2, path analyses indicated that BMI did not have direct effect on HbA1c. Mediating effects of BMI on the Apo-HbA1c association were not found in subjects (Fig. 2).

## Discussion

To the best of our knowledge, the present study firstly identified the potential influence of ApoA1, ApoB concentrations and the ApoB/A1 ratio on HbA1c level in middle-aged and elderly Chinese populations. Our results showed that a higher serum ApoA1 concentration was associated with lower HbA1c level, while higher ApoB concentration and ApoB/A1 ratio were associated with higher HbA1c level. There was no evidence of the mediating effects of BMI on the Apo-HbA1c associations.

### ApoA1 and HbA1c level

ApoA1, a major protein component of HDL, has been shown to have a distinct effect on glucose metabolism^22^. The protective effect of HDL was partly attributed to ApoA1^23^. Our results demonstrated that both ApoA1 and an intermediate HDL-C levels were inversely associated with HbA1c. An in vivo and in vitro study showed that ApoA1 could improve glucose metabolism by attenuating ability of catalyzing cholesterol efflux alter protein structure and decreasing lipid binding capability^24^. This effect was confirmed in a cross-sectional study involving 137 women and 111 men^12^, Sosenko et al*.* reported that ApoA1 was inversely related to HbA1c level in both men and women (r_women_ = − 0.25 and r_men_ = − 0.30). Nevertheless, in a cross–sectional study with 17,661 participants within the EPIC–Norfolk cohort study, Julian et al*.* found null associations between serum ApoA1 and HbA1c level^25^. Likewise, Boris et al*.* and Schauer et al*.* also reported that ApoA1 was not related to HbA1c level^13,14^. Some factors might provide explanation for these discrepancies. The favorable association was more readily to be observed in persons with higher HbA1c level (5.69% [this study] and 6.37%^12^ vs. 5.3%^25^), in large sample size (1448 subjects [this study] vs. 44^13^ and 238 subjects^26^). Lipid values formed part of the inclusion criteria, intensity of glycemic therapy and residual insulin secretion might also limit variability in Apo in an early type 2 diabetic population^14^. More large prospective studies are needed to confirm our results. Other reasons, such as the differences in the adjusted covariates and analysis method (e.g., multiple regression analyses^12^, student's t-test^13^, partial correlations^14^, linear regression analyses^25,26^ and analyses of covariance [this study]), might also explain the differences in these studies.

### ApoB, ApoB/A1 ratio and HbA1c level

ApoB is the main lipoprotein associated with LDL and lipoprotein(a) particles^27^. Our study observed that ApoB, but not LDL-C was positively associated with HbA1c. Ley et al*.* pointed out that plasma ApoB was superior to LDL-C in predicting the diseases in Canadian population^28^. There is growing interest in the health benefits of ApoB. Although previous studies have shown the unfavorable roles of ApoB and the ApoB/A1 ratio in the cerebrovascular diseases and diabetes^5,29^, scarce data assessed the associations of ApoB level and ApoB/A1 ratio with HbA1c. Based on data from 44 diabetics who participated in the Erfurt Study, higher ApoB level was observed in HbA1c ≥ 8% group than in HbA1c < 8% group^13^. Consistent with this finding, we also observed the positive associations of ApoB and the ApoB/A1 ratio with HbA1c in Chinese adults. Nevertheless, Mustapha et al*.*^26^ found no significant differences in ApoB/A1 ratio among different HbA1c groups in both men and women with type 2 diabetes. Hypoglycemic drugs using might attenuate the association between ApoB/A1 ratio and HbA1c in 238 type 2 diabetic patients^26^. Additionally, previous study pointed out that ApoB/A1 ratio exerted significant information for predicting insulin resistance^30^. Our study also indicated that ApoB/A1 ratio might act as a more precise marker for HbA1c measures (ApoA1: (Q4–Q1)/Q1 = − 0.021; ApoB: (Q4–Q1)/Q1 = 0.039; ApoB/A1 ratio: (Q4–Q1)/Q1 = 0.046).

### Potential mechanisms

The associations of ApoA1 and ApoB with HbA1c might be explained by various mechanisms. First, ApoA1 may improve glucose tolerance by adenosine monophosphate-activated protein kinasecomplex and increase glucose uptake into skeletal muscle and heart^31^. In addition, ApoA1 can increase insulin sensitivity in skeletal muscle and adipose tissue^10^ and reduce lipid binding capability^24^. Second, dysregulation of ApoB metabolism can induce insulin resistance^32^ and inhibit lipolysis from liver to peripheral fat via acting as a lipid metabolic pathway^33^. However, our path analysis showed no mediating effects of BMI on Apo-HbA1c association. Other inflammation and oxidative stress markers might be mediated in the associations of ApoA1 and ApoB with HbA1c. Finally, ApoA1 can inhibit inflammation via decreasing plasma malondialdehyde level and clearing pro-inflammatory lipids^10^. ApoB can aggravate inflammation by releasing inflammatory cytokines (e.g., IL-1β and tumor necrosis factor-α)^34^ and binding to enolase-1. Nevertheless, the detailed mechanism underlying the associations of ApoA1 and ApoB with HbA1c remains unclear and warrants further investigation.

### Strengths and limitations

This study has several strengths. Primarily, to the best of our knowledge, this is the first report that evaluated the associations of serum ApoA1, ApoB and the ApoB/A1 ratio with HbA1c in both cross-sectional and prospective analyses, and examined the mediating effects of BMI on Apo-HbA1c association in Chinese adults. Next, the relatively large sample size provided us with sufficient power to evaluate potential associations among variables. Third, the availability of individual information (e.g., basic characteristics, lifestyles and medication records) allowed us to avoid potential confounding effects. Finally, the probability of falsely significant founds was reduced by assessing different Apo and lipid indices. Notwithstanding, our study also had a few limitations. First, the lack of association between Apo and HbA1c changes in our longitudinal analyses. This might partly be explained by the observed small longitudinal changes in HbA1c [mean (± SEM) HbA1c changes: − 0.030 ± 0.024], which accounted for < 1% of the mean HbA1c. The small changes in HbA1c would also substantially attenuate the Apo-HbA1c association, particularly in the longitudinal studies. The effect size might be substantially underestimated in the present study, and that is the reason (at least in part) why the Apo-HbA1c associations were much more significant in the cross-sectional data than in their longitudinal counterparts. Thus, the effects of Apo need to be clarified in further prospective studies. Second, the effects of dietary nutrients were not excluded, which might attenuate the underlying associations due to dietary nutrients were associated with Apo concentrations^35^. Third, as with any observational study, residual confounding could not be ruled out due to the potential confounders. Finally, we could not rule out the possibility of selection bias because our study subjects were not a random sample of Chinese community population. However, we did not found any significant interactions between Apo and age, sex, exercise, smoking, or alcohol drinking on HbA1c. The generalizability of the findings was unlikely to be influenced by these factors.

## Conclusion

Our cross-sectional findings suggested that higher ApoA1, lower ApoB concentrations and the ApoB/A1 ratio were associated with lower HbA1c level in Chinese adults. More large prospective studies with the longer follow-up period are needed to clarify the effect of Apo on HbA1c changes.

## Supplementary Information

Supplementary Tables.

## Data Availability

All data generated or analysed during this study are included in this published article (and its Supplementary Information files).
