# Supplementary material for: Cross-sectional and longitudinal associations of apolipoprotein A1 and B with glycosylated hemoglobin in Chinese adults
Source: Sci Rep. 2022 Feb 17;12:2751. doi: 10.1038/s41598-022-06829-w (PMC8854380; doi:10.1038/s41598-022-06829-w)
Supplement: Supplementary file 1 — Supplementary Tables. [file 41598_2022_6829_MOESM1_ESM.doc]

**Supplementary Tables 1.** Mean HbA1c level according to quartiles of lipids in all participants (Mean ± SE)

| **Variables** |  | **Quartiles by Apolipoproteins** | | | | ***P*-Diff** | ***P*-Trend** |
| --- | --- | --- | --- | --- | --- | --- | --- |
|  |  | **Q1** | **Q2** | **Q3** | **Q4** |  |  |
| TG |  |  |  |  |  |  |  |
| n |  | 359 | 361 | 364 | 364 |  |  |
| HbA1c | Model 1 | 5.633±0.034 | 5.686±0.034 | 5.655±0.034 | **5.774±0.034^a^** | **0.019** | **0.010** |
|  | Model 2 | 5.629±0.034 | 5.675±0.034 | 5.653±0.034 | 5.790±0.034 | **0.004** | **0.002** |
|  | Model 3 | 5.635±0.033 | 5.691±0.033 | 5.650±0.033 | **5.772±0.033^ac^** | **0.016** | **0.014** |
| TC |  |  |  |  |  |  |  |
| n |  | 364 | 363 | 361 | 360 |  |  |
| HbA1c | Model 1 | 5.644±0.034 | 5.661±0.034 | 5.661±0.034 | **5.783±0.034^a^** | **0.015** | **0.006** |
|  | Model 2 | 5.659±0.034 | 5.668±0.034 | 5.653±0.034 | 5.770±0.034 | **0.046** | **0.036** |
|  | Model 3 | 5.660±0.033 | 5.654±0.033 | 5.656±0.033 | **5.778±0.033^b^** | **0.019** | **0.017** |
| LDL-C |  |  |  |  |  |  |  |
| n |  | 366 | 356 | 362 | 364 |  |  |
| HbA1c | Model 1 | 5.658±0.034 | 5.669±0.035 | 5.693±0.034 | 5.728±0.034 | 0.485 | 0.127 |
|  | Model 2 | 5.667±0.034 | 5.683±0.034 | 5.683±0.034 | 5.716±0.034 | 0.765 | 0.323 |
|  | Model 3 | 5.658±0.033 | 5.678±0.033 | 5.686±0.033 | 5.726±0.033 | 0.530 | 0.153 |
| HDL-C |  |  |  |  |  |  |  |
| n |  | 368 | 353 | 365 | 362 |  |  |
| HbA1c | Model 1 | 5.763±0.034 | 5.671±0.035 | 5.642±0.034 | 5.672±0.034 | 0.068 | **0.049** |
|  | Model 2 | 5.769±0.033 | 5.666±0.034 | **5.641±0.034^a^** | 5.671±0.034 | **0.037** | **0.034** |
|  | Model 3 | 5.763±0.033 | 5.667±0.033 | **5.639±0.033^a^** | 5.678±0.033 | **0.047** | 0.058 |

Abbreviations were shown in Tables 1 and 2. *P*-Diff: Multiple comparison among quartiles. Model 1 was univariate analysis. Model 2 was adjusted for sex, age. Model 3 further adjusted for BMI, education, marriage, exercise, cigarette smoking, alcohol consumption, hypoglycaemic agent, and lipid-lowering drugs use. ^a^*P* < 0.05 compared with Q1. ^b^*P* < 0.05 compared with Q2. ^c^*P* < 0.05 compared with Q3.

**Supplementary Tables 2.** Changes in HbA1c level over ~4 y by quartiles of lipids in all participants (Mean ± SE)

| **Variables** |  | **Quartiles by Apolipoproteins** | | | | ***P*-Diff** | ***P*-Trend** |
| --- | --- | --- | --- | --- | --- | --- | --- |
|  |  | **Q1** | **Q2** | **Q3** | **Q4** |  |  |
| TG |  |  |  |  |  |  |  |
| n |  | 204 | 210 | 205 | 207 |  |  |
| HbA1c | Model 1 | 0.010±0.048 | 0.048±0.047 | -0.077±0.048 | -0.102±0.047 | 0.082 | **0.030** |
|  | Model 2 | 0.011±0.048 | 0.047±0.047 | -0.077±0.048 | -0.102±0.047 | 0.084 | **0.030** |
|  | Model 3 | -0.001±0.048 | 0.040±0.047 | -0.076±0.048 | -0.083±0.048 | 0.197 | 0.093 |
| TC |  |  |  |  |  |  |  |
| n |  | 205 | 208 | 206 | 207 |  |  |
| HbA1c | Model 1 | -0.058±0.048 | -0.051±0.047 | -0.032±0.048 | 0.020±0.048 | 0.653 | 0.238 |
|  | Model 2 | -0.057±0.048 | -0.051±0.047 | -0.032±0.048 | 0.019±0.048 | 0.662 | 0.247 |
|  | Model 3 | -0.059±0.048 | -0.051±0.047 | -0.025±0.047 | 0.015±0.047 | 0.688 | 0.244 |
| LDL-C |  |  |  |  |  |  |  |
| n |  | 207 | 207 | 205 | 207 |  |  |
| HbA1c | Model 1 | -0.072±0.047 | -0.048±0.047 | 0.018±0.048 | -0.017±0.047 | 0.576 | 0.281 |
|  | Model 2 | -0.071±0.048 | -0.048±0.048 | 0.017±0.048 | -0.018±0.048 | 0.592 | 0.290 |
|  | Model 3 | -0.070±0.047 | -0.046±0.047 | 0.019±0.048 | -0.022±0.047 | 0.591 | 0.324 |
| HDL-C |  |  |  |  |  |  |  |
| n |  | 206 | 213 | 201 | 206 |  |  |
| HbA1c | Model 1 | -0.093±0.048 | -0.052±0.047 | -0.001±0.048 | 0.029±0.048 | 0.281 | 0.051 |
|  | Model 2 | -0.093±0.048 | -0.052±0.047 | -0.002±0.048 | 0.029±0.048 | 0.278 | 0.051 |
|  | Model 3 | -0.084±0.048 | -0.051±0.047 | 0.001±0.048 | 0.017±0.048 | 0.426 | 0.101 |

Abbreviations were shown in Table 1 plus SE: standard error and Q: quartile. *P*-Diff: Multiple comparison among quartiles. Model 1 was univariate analysis. Model 2 was adjusted for sex, age. Model 3 further adjusted for BMI, education, marriage, exercise, cigarette smoking, alcohol consumption, hypoglycaemic agent, and lipid-lowering drugs use.
